# Status and factors associated with patient activation and its relationship with HIV clinic outcomes among Yi minority people living with HIV in Liangshan, China: a cross-sectional study

**DOI:** 10.3389/fpubh.2023.1114561

**Published:** 2023-06-15

**Authors:** Wenhong An, Xuefeng Tang, Xueling Xiao, Waha Aku, Honghong Wang

**Affiliations:** ^1^Xiangya School of Nursing, Central South University, Changsha, China; ^2^Sichuan Center for Disease Control and Prevention, Chengdu, Sichuan, China; ^3^Red Ribbon Antiviral Care Center, Zhaojue County People's Hospital, Liangshan, China

**Keywords:** HIV, patient activation, illness perception, Yi minority, low-and middle-income

## Abstract

**Introduction:**

Patient activation is determined by an individual’s knowledge, skills and confidence in managing his/her health. It is vital for people living with HIV (PLWH) to enhance their self-management skills and health outcomes, especially those from low- and middle-income regions, since they are at higher risk of worse health outcomes. However, literature from those regions is limited, especially in China.

**Objectives:**

This study aimed to explore the status and factors associated with patient activation among Yi minority PLWH in Liangshan, China and to determine whether patient activation is associated with HIV clinic outcomes.

**Methods:**

This cross-sectional study included 403 Yi minority people living with HIV in Liangshan between September and October 2021. All participants completed an anonymous survey measuring sociodemographic characteristics, HIV-related information, patient activation and illness perception. Multivariate linear regression and multivariate binary logistic regression were used to explore factors associated with patient activation and the association between patient activation and HIV outcomes, respectively.

**Results:**

The Patient Activation Measure (PAM) score was low (mean = 29.8, standard deviation = 4.1). Participants with negative illness perception, low income, and self-rated antiretroviral therapy (ART) effect based on self-perception were most likely to have a lower PAM score (β = −0.3, −0.2, −0.1, respectively; all *p* < 0.05); those with having disease knowledge learning experiences and an HIV-positive spouse were more likely to have a higher PAM score (β = 0.2, 0.2, respectively; both *p* < 0.001). A higher PAM score (AOR=1.08, 95% CI: 1.02, 1.14) was associated with viral suppression, mediated by gender (AOR=2.25, 95% CI: 1.38, 3.69).

**Conclusion:**

Low patient activation level among Yi minority PLWH impacts HIV care. Our findings indicate patient activation is associated with viral suppression for minority PLWH in low- and middle-income settings, suggesting that tailored interventions enhancing patient activation may improve viral suppression.

## Introduction

As the prevalence of human immunodeficiency virus (HIV) infection steadily increases, it remains one of the most challenging health issues in the world, particularly in low- and middle-income countries. Globally, more than 37 million people suffer from HIV infection, and over two-thirds of them (25.4 million) are in the low-and middle-income regions (1). The number of people living with HIV (PLWH) in China is approximately 1.05 million (2). Liangshan Yi Autonomous Prefecture (hereafter, Liangshan) is the largest settlement of Yi minority people; it is a region with the most serious HIV endemic (3) and the poorest economic status in comparison to other places in China (4). In 2020, it was reported Liangshan had 43,697 PLWH (4). This population was more likely to suffer from worse clinical outcomes than their counterparts from other regions, since in Liangshan, fewer medical resources are available and fewer HIV education programs are provided in the Yi language, resulting in suboptimal self-management (5).

As antiretroviral treatment (ART) became widely available, HIV/AIDS has become a chronic illness, and self-management of HIV-related issues is vital for patients to achieve and maintain viral suppression to facilitate optimal quality of life. Patient activation is determined by an individual’s knowledge, skills and confidence in managing his/her health; this is essential for self-management and health outcomes as it enables patients to actively participate in their healthcare (6–8). According to a study conducted by Hibbard and colleagues (9), patient activation is changeable and includes four stages, ranging from low (Level 1) to high (Level 4).

In terms of associated factors, previous studies have confirmed that sociodemographic factors, including educational level, income level, age, gender and disease-related factors, such as illness perception and health status, have an impact on patient activation in chronic disease patients (8, 10–13). Among these factors, illness perception appears to be a driving force for patient activation (14). Different levels of illness perception can lead to negative or positive coping styles, and patients with low patient activation tend to be passive and are less engaged in their own care, while those with high patient activation are actively involved in their care (15). However, to the best of our knowledge, the correlation between illness perception and patient activation in PLWH has not yet been studied.

Current studies about patient activation have focused on chronic diseases that are not communicable (e.g., chronic obstructive pulmonary disease and diabetes) (16); Previous studies on patient activation in PLWH from high-income countries have shown that the patient activation level is high and is associated with HIV clinical outcomes, such as CD4 count, viral suppression and adherence (13, 17). However, there is a lack of studies reporting the status and factors associated with patient activation and its relationship with HIV clinic outcomes among PLWH from low- and middle-income countries. Given the persistent differences in cultures, social status, economic health and healthcare delivery systems, it is expected that there will be differences in patient activation between low- and middle-income countries and high-income countries (18). In view of the literature gap mentioned above, we decided to conduct the present study. Thus, in this study, we selected the Yi minority PLWH from Liangshan, a low-income area in China, as the study subject. Based on these premises, the objectives in this study were twofold: First, to explore the status and factors associated with patient activation among Yi minority PLWH in Liangshan, China. Second, to determine whether patient activation is associated with HIV clinic outcomes.

## Materials and methods

### Study design, setting, and sample

This cross-sectional study was conducted in the main HIV clinic located in Liangshan Yi Autonomous Prefecture, which is a region with the most serious HIV endemic and the poorest economic status in comparison to other places of China. In previous studies, the prevalence of HIV infections has been found to be higher (2.88–9.46%) in Yi minority people in comparison to the Chinese average (0.04%) (19, 20). In addition to providing routine HIV care, this clinic offers a brief peer-based HIV knowledge education program in the Yi language, which is held once a week for half an hour. Thus, only a few patients have the opportunity to attend the program. To be clear, the content of this program just includes basic knowledge of HIV/AIDS. Details are as follows: (1) What is HIV? (2) How does HIV spread? (3) How does HIV infection progress? (4) Why do alternative treatments work for HIV?

The participants were PLWH seeking care at this clinic, such as obtaining ART medication and undergoing lab tests for viral load and CD4 counts. The inclusion criteria were: having a confirmed HIV diagnosis, being 18 years of age or older, being a member of the Yi minority group and currently receiving antiviral therapy. PLWH who were unable to understand the questionnaire because of cognitive or audio-visual impairment were excluded from participating in this study.

The consecutive sampling method was applied to recruit the participants when they came to the clinic for services. PASS software was used to estimate the sample size. Based on a previous study (21), the standard deviation of the patient activation mean raw score was estimated to be 5.67. In the present study, the sample size of 341 participants was estimated based on the statistical power of 95% and the alpha value of 0.05. Considering that 15% of the questionnaires were invalid, the final sample size was 392 participants.

### Measurements

In this study, the variables included patient activation, illness perception, ART adherence and sociodemographic and disease-related information. The data collection instruments included the 13-item Patient Activation Measure (PAM), the Brief Illness Perception Questionnaire (BIPQ), the Center for Adherence Support Evaluation (CASE) Adherence Index and a sociodemographic and disease-related information questionnaire, as described below.

#### Patient activation

PAM was used to determine the level of patient activation, and we obtained permission from Insignia Health to use this scale. It contains 13 items ranging from strongly disagree (1) to strongly agree (4) on a 4-point Likert scale [9]. Using a revised transformation table provided by Insignia Health, raw scores, in the range of 13 to 52, are calibrated into a total score between 0 and 100 (22). In a previous study, the PAM raw scores clearly showed differences in patient activation in comparison to transformed scores (21). Thus, our study used PAM raw scores instead of transformed scores. Higher scores indicate higher levels of patient activation. The developers of PAM classified the scores into four levels (23). Level 1 patients (raw score ≤ 35.9) are passive and lack the confidence needed to actively participate in their health care. Level 2 patients (raw score 35.91–38.6) have some knowledge, but are starting to gain more knowledge and feel more confident about managing their condition; while large gaps remain, they can set simple goals. Level 3 patients (raw score 38.7–42.5) are goal-oriented, have key facts, are developing skills and are prepared to act. Level 4 patients (raw score ≥ 42.6) have adopted new behaviors for managing their health but may have problems when under stress (21). PAM has been translated into Chinese and has shown satisfactory reliability and validity. Cronbach’s α was 0.82, test–retest reliability was 0.70 and the construct validity was favorable in a Chinese population, as confirmed by factor analysis (24).

#### Illness perception

Measures of illness perception were obtained using Broadbent and colleagues’ Brief Illness Perception Questionnaire (BIPQ), developed in 2006 (15). The BIPQ is an 11-point (0–10) Likert response scale; it contains 8 items. Items 3, 4 and 7 are scored reversely when calculating the total score. A higher total score, which ranges from 0 to 80, indicates a greater negative illness perception of the disease. In the first five items of this scale, consequences, duration, personal control, treatment control and identity are examined; two items, anxiety and emotional burden, are related to affective visualization; one item assesses the participants’ understanding of their disease. A Chinese version of this questionnaire was found to be valid and reliable by Mei (25). Its Cronbach’s α was 0.77 and its criterion-related validity was −0.67.

#### ART adherence

The CASE Adherence Index was used to measure adherence to ART (26). It consists of three questions. One question asks the respondents to note the difficulty they experience in taking their HIV medication on time. The responses include always, most of the time, rarely or never (score ranging from 1 to 4). The second question asks the respondents to note how many days per week an HIV medication dose has been missed; its answers are every day, 4–6 days, 2–3 days, once a week, less than once a week or never (score ranging from 1 to 6). The third question asks the respondents to note the last time that they missed at least one HIV medication dose. Answers include within the past week, one to two weeks ago, three to four weeks ago, one to three months ago, more than three months ago or never (score range of 1 to 6). The maximum score of the CASE Adherence Index is 16; a higher score indicates better adherence. A score equal to or greater than 11 is considered good adherence and a score less than 11 indicates poor adherence.

#### Sociodemographic and disease-related information

A self-made sociodemographic questionnaire was developed to collect data on gender, age, education, marital status, place of residence, employment status and personal income. Disease-related information was also assessed in terms of disease duration, disease knowledge learning experience, spouse’s HIV status, HIV viral load suppression and CD4 count and the self-rated method of ART effect. The question “Have you ever received previous health education about HIV/AIDS (such the program offered by the clinic, or other similar programs)?” was used to measure the disease knowledge learning experience; and the answers included “Yes” and “No.” This variable refers to the experience in taking part in the peer-based HIV education program offered by the clinic, or other similar programs. It is well known that HIV viral load and CD4 count are crucial markers for evaluating the efficacy of ART. Despite this, most of the Yi minority PLWH were unaware of their own CD4 count and HIV viral load based on field observations. Hence, to explore the self-rated method of the ART effect, we devised a question: How do you judge the ART effect? Answers to this question included: (a) by HIV viral load and CD4 count or (b) by self-perception (such as no pain, have a good appetite and no hospitalization).

### Data collection

We found that most of the Yi minority PLWH could not read Mandarin; therefore, we conducted face-to-face interviews using the questionnaire instead of using the self-reported questionnaires. The questionnaires were translated and back-translated independently by two experts (a researcher and a Yi language expert) who are fluent in both Yi language and Mandarin; they were then assessed for cultural relevance and understandability. Data were collected from September to October 2021 by four trained interviewers fluent in both Yi language and Mandarin. All of the interviewers received face-to-face training on the purpose and design of the study, interviewing techniques and role-playing to minimize the interrater differences in using the questionnaires. The participants were approached when they came to the clinic to pick up their medications or receive other medical services.

Physicians introduced our project to patients and referred them to a trained investigator for screening and recruitment. A private and quiet room was provided for data collection. The interviewing process usually took between 15 and 25 min for each candidate. Information on the HIV viral load and CD4 count were extracted from each patient’s medical records with permission for the participants and the physicians.

### Data analysis

Descriptive statistics were used to summarize the characteristics of the sample. Mean and standard deviation (SD) were used to describe the continuous variables, and frequencies and percentages were used to summarize the categorical variables. Bivariate associations were analyzed using Spearman’s rank correlation coefficient (*r_s_*), the Kruskal–Wallis test or the Mann–Whitney test, depending on the type of data. Multivariate linear regression analysis was conducted on the variables related to patient activation at a significance level of *p* < 0.05 for the univariate analysis.

To test the association between patient activation and HIV viral suppression and CD4 count, univariate and multivariate binary logistic regression analyses were used. The association between patient activation and viral suppression has previously been demonstrated to be mediated by ART adherence (13). Therefore, adherence and other variables that were statistically significant (*p* < 0.05) in the univariate analysis were adjusted in the multivariable logistic regression analysis of patient activation and viral suppression. Thus, *p* < 0.05 (two-sided) was considered significant for the final multivariate analysis. SPSS 23.0 (IBM Corporation, Armonk, NY, United States) was used for statistical analyses.

## Results

### Sociodemographic and disease-related characteristics

Among the 409 participants recruited, 403 (98.5%) completed the questionnaire. Table 1 shows that the mean age of the participants was 40.1 years, ranging from 18 to 77 years of age. Most of the participants were male (*n* = 238, 59.1%), married (*n* = 307, 76.2%) and residing in rural areas (*n* = 386, 95.8%). Educational attainment was fairly low with 93.5% (*n* = 377) reporting a primary level education or less. Most of the participants were farmers (*n* = 340, 84.4%) and most had a personal monthly income of less than US$138 (*n* = 271, 67.2%). Among the 403 participants, 87.3% (*n* = 352) reported self-rated method of ART effect based on self-perception and 86.1% (*n* = 347) having disease knowledge learning experience. Approximately half of the patient’s spouses (*n* = 203, 50.4%) were also HIV positive. The average disease duration was 7.3 years. The mean BIPQ score was 33.33 (SD 7.2, range 10–61). The mean CASE score was 14.7 (SD 2.1, range 5–16).

**Table 1:** Sociodemographic and disease-related characteristics and the univariate analysis of patient activation for Yi minority PLWH (*n* = 403).

Characteristics	Mean ± SD/N (%)	PAM	*Z*/*H*/*r_s_*	*p*
Age (years)	40.1 ± 8.2	29.8 ± 4.1	*r_s_ =* −0.07	0.148
Gender			*Z =* −1.7	0.094
Male	238 (59.1)	29.8 ± 3.8		
Female	165 (40.9)	29.8 ± 4.5		
Resident place			*Z =* −1.4	0.158
Rural	386 (95.8)	29.9 ± 4.0		
Urban	17 (4.2)	28.0 ± 5.1		
Education level			*H =* 1.8	0.455
Primary school or below	377 (93.5)	29.8 ± 4.1		
Middle school	20 (5.0)	29.4 ± 3.6		
High school or above	6 (1.5)	31.5 ± 3.9		
Marital status			*H =* 1.1	0.770
Unmarried	25 (6.2)	30.7 ± 2.6		
Married	307 (76.2)	29.8 ± 4.1		
Divorced	15 (3.7)	30.7 ± 2.6		
Widowed	56 (13.9)	29.3 ± 4.7		
Employment status			*H =* 2.6	0.270
Full-time	43 (10.7)	30.3 ± 4.0		
Part-time	20 (5.0)	29.6 ± 2.6		
Farming	340 (84.4)	29.7 ± 4.2		
Disease duration (years)	7.3 ± 3.9		*r_s_ = 0.03*	0.529
Personal monthly income			*H =* 23.8	< 0.001
≤ US$138	271 (67.2)	29.7 ± 4.3		
US$139 to US$414	61 (15.1)	28.6 ± 3.5		
≥ US$415	71 (17.6)	31.3 ± 2.9		
Self-rated method of ART effect			*Z =* −4.1	< 0.001
Viral load and CD4 count	51 (12.7)	31.4 ± 4.8		
Self-perception	352 (87.3)	29.6 ± 3.9		
Having disease knowledge learning experience			*Z = −*3.1	0.001
Yes	347 (86.1)	30.1 ± 3.9		
No	56 (13.9)	28.0 ± 4.9		
Spouse’s HIV status			*H =* 13.0	0.001
HIV negative	175 (43.4)	29.0 ± 4.7		
HIV positive	203 (50.4)	30.4 ± 3.5		
Other (unmarried)	25 (6.2)	30.7 ± 2.6		
Illness perception	33.3 ± 7.2	29.8 ± 4.1	*r_s_ =* −0.3	< 0.001
Adherence	14.7 ± 2.1	29.8 ± 4.1	*r_s_ =* −0.09	0.083

SD, standard deviation; PAM, patient activation measure.

### Patient activation status

**Figure 1:**
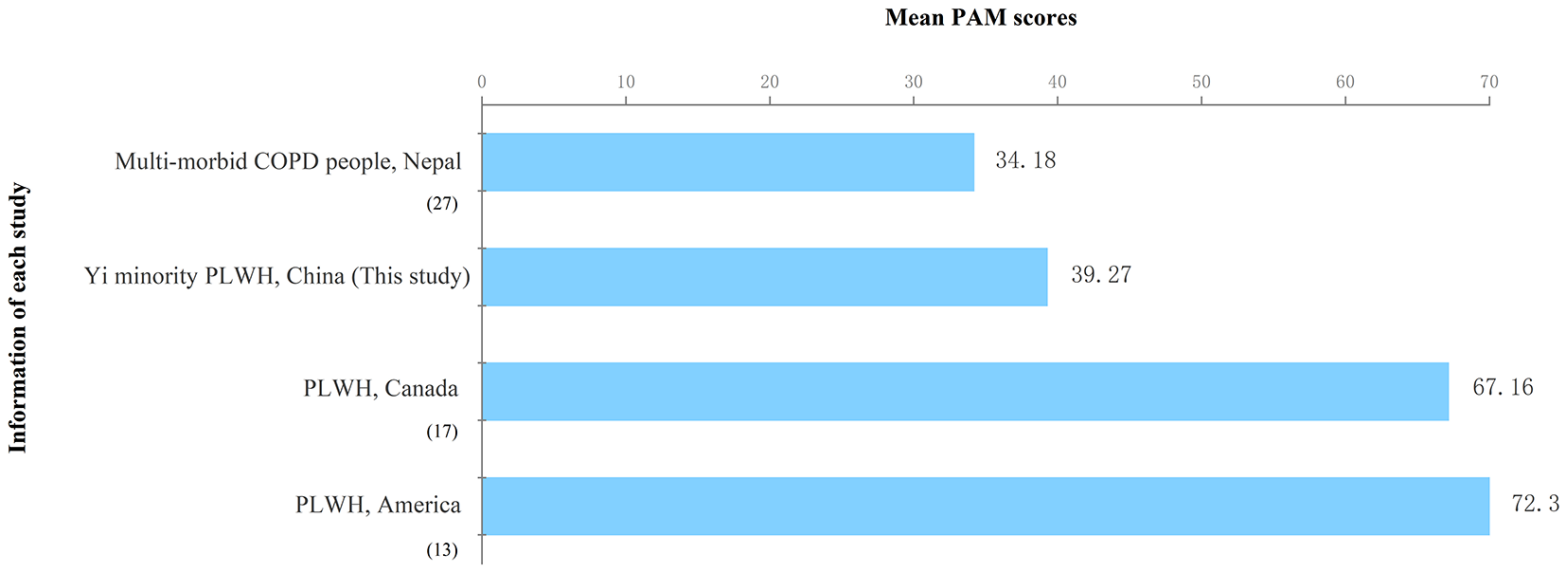
Comparison of the current study’s mean PAM score with the scores reported in previous studies.

The PAM raw score was 29.8 ± 4.1 with a range of 14–45 (corresponding to the transformed score: 39.3 ± 6.0). A comparison of results from this study and previous studies (13, 17, 27) is shown in Figure 1. In terms of patient activation level, the percentage of participants in Level 1 was as high as 97.8% (*n* = 394); the percentage of patients in Level 2 and Level 3 were 1.7% (*n* = 7) and 0.5% (*n* = 2), respectively. There were no Level 4 cases in this survey.

### Univariate and multivariate analyses of factors associated with patient activation

Table 1 presents the relationships between the study variables and patient activation. Regarding the demographic characteristics, a significant correlation was found between personal monthly income (*H* = 23.8, *p <* 0.001) and PAM scores among Yi minority PLWH. For the disease-related variables, the self-rated method of ART effect (*Z =* −4.1, *p* < 0.001), having disease knowledge learning experience (*Z* = −3.1, *p =* 0.001), spouse’s HIV status (*H* = 13.0, *p =* 0.001) and illness perception (*r_s_ =* −0.3, *p* < 0.01) were significantly associated with the PAM score.

The results from the multiple linear regression analysis of patient activation are presented in Table 2. Before conducting the analysis, the categorical variables were transformed into dummy variables. Multicollinearity was checked and was not present (all VIF < 2).

**Table 2:** Multiple linear regression analysis of the variables in relation to patient activation.

Variables	SD	*β*	*t*	*p*	VIF
Having disease knowledge learning experience
No	Ref				
Yes	0.5	0.2	3.9	< 0.001	1.0
Personal monthly income					
≤ US$138	Ref				
US$139 to US$414	0.6	−0.2	−3.7	< 0.001	1.6
≥ US$415	0.5	−0.1	−2.6	0.010	1.6
Self-rated method of ART effect
Viral load and CD4+ counts	Ref				
Self-perception	0.6	−0.1	−2.4	0.016	1.0
Spouse’s HIV status					
HIV negative	Ref				
HIV positive	0.4	0.2	4.4	< 0.001	1.0
Other (unmarried)	0.8	0.1	1.7	0.098	1.0
Illness perception	0.03	−0.3	−7.3	< 0.001	1.0

SE, standard error; VIF, variance inflation factor; Ref, reference.

The findings indicate that the participants with having disease knowledge learning experience (*β* = 0.2, *p* < 0.001) and whose spouse was HIV positive (*β* = 0.2, *p* < 0.001), had a significantly higher PAM score. The participants were more likely to have a lower PAM score if their personal monthly income ranged from US$139 to US$414 (*β* = −0.2, *p* < 0.001) or was ≤ US$138 (*β* = −0.1, *p* = 0.010), if their self-rated method of ART effect based on self-perception (*β* = −0.1, *p* = 0.010) and if they had a higher illness perception score (*β* = −0.3, *p* < 0.001).

### The relationship between patient activation and HIV clinical outcomes

Of the 403 participants, 387 reported HIV clinical outcomes in terms of CD4 count and viral suppression (HIV viral load <50 copies/mL). Among these, 280 (72.4%) participants achieved viral suppression and 326 (84.2%) achieved a CD4 count ≥200 cells/mL^3^. Univariate logistic regression analysis revealed that there was no significant association between patient activation and CD4 count (odds ratio [OR] = 1.01, 95% confidence interval [CI]: 0.94, 1.08, *p* = 0.771), while it was positively related to viral suppression (OR = 1.09, 95% CI: 1.03, 1.15, *p* = 0.002).

Table 3 reports the univariable and multivariable associations between viral suppression and patient activation. The univariable regression showed significant associations between viral suppression and patient activation, gender and illness perception. After adjusting for gender and illness perception, patient activation (AOR = 1.08, 95% CI: 1.02, 1.14, *p* = 0.011) remained positively related to viral suppression, and the relationship was mediated by gender (AOR = 2.25, 95% CI: 1.38, 3.69, *p* = 0.001). However, ART adherence was not significantly related to viral suppression.

**Table 3:** Univariable and multivariable logistic regression analysis of patient activation and viral suppression (*n* = 387).

Variables	Viral suppression			
	Crude OR (95% CI)	*p*	Adjusted OR (95% CI)	*p*
Gender
Male	Ref			
Female	2.16 (1.34, 3.48)	0.002	2.25 (1.38, 3.69)	0.001
Adherence	1.08 (0.97, 1.20)	0.146	1.05 (0.95, 1.17)	0.333
Illness perception	0.97 (0.94, 1.00)	0.050	0.98 (0.94, 1.01)	0.183
Patient activation	1.09 (1.03, 1.15)	0.002	1.08 (1.02, 1.14)	0.011

OR, odds ratio; 95% CI, 95% confidence intervals; Ref, reference.

## Discussion

To the best of our knowledge, this is the first study to explore the status of patient activation and its correlation with health outcomes (CD4 count, viral suppression) in Yi minority PLWH in China. We found three key findings in our research. First, the PAM score was extremely low, which is contrary to the findings reported in previous research conducted in high-income countries. Second, lower personal monthly income, self-rated method of ART effect based on self-perception and a higher illness perception score were associated with a lower PAM score. Having disease knowledge learning experience and spouse’s positive HIV status were related to a higher PAM score. Third, a higher PAM score was related to viral suppression, mediated by gender. To achieve optimal HIV self-management, these findings are critical to improving patient activation in low- and middle-income settings.

In this study, patient activation status was found to be different from that reported in previous studies. The level of PAM in Yi minority PLWH is much lower than in studies conducted in Western countries, such as the United States (13) and Canada (17). It is concerning that more than 90% of our sample have Level 1 PAM scores. Our results are similar to those reported in a study from Nepal, which showed that 81.5% of the sample were at patient activation Level 1 (27, 28). The lack of patient activation education programs in the Yi minority language could be an important reason for this finding. In our study, the level of education for most of the participants was primary school or less; thus, they have barriers to accessing HIV education programs in Mandarin. Moreover, in the health service system of Liangshan the official language is Mandarin. Although some healthcare providers can speak the Yi language, communication barriers still exist between Yi minority PLWH and healthcare providers. It is common for patients with low PAM scores to have a lack of understanding of their roles in the healthcare process and they have low confidence in their self-management skills (8). Consequently, they are less likely to consult with their providers when needed, which may result in poor HIV management, increasing the chances of hospitalization and high mortality (7). Therefore, it is necessary to conduct further research on tailored patient activation interventions for minority PLWH in low- and middle-income regions.

We found that lower personal monthly income, self-rated method of ART effect based on self-perception and higher illness perception score were associated with a lower PAM score. It is not surprising that lower personal monthly income is a risk factor for a low PAM score, which is consistent with the findings reported in previous studies that the income levels of participants are related to patient activation (29–31). Participants with a lower income might spend less money and exert less effort on health maintenance (31), resulting in a low PAM score; they may also adopt more passive behavior toward the management of their health. In our study, the self-rated method of ART effect based on self-feeling rather than CD4 count or viral load was related to a lower PAM score. This finding suggests that participants lack the proper information to monitor the effect of their treatment. They neither understand the purpose of the blood tests nor know the meaning of the values of their CD4 counts and viral load. It is well known that information is a necessary prerequisite for acquiring the skills needed to manage one’s health (32). Furthermore, we found that a higher illness perception score, which represents greater negative reactions to the disease, was a significant predictor of a lower PAM score. This finding was also supported by previous studies (10, 33, 34); it could be explained by the fact that different levels of illness perception can lead to negative or positive coping styles (patients with low patient activation tend to be passive and those with high patient activation tend to have positive coping styles) (15, 35).

Having disease knowledge learning experience and a spouse’s positive HIV status were predictors of a higher PAM score. In this context, the PLWH with having disease knowledge learning experience from a brief peer-based HIV knowledge education program organized weekly in the clinic, but part of the sample did not have the opportunity to participate in this program. According to several previous studies of chronic disease patients, providing patients with knowledge about their disease can significantly improve the PAM score (7, 36). We believe that having disease knowledge and learning experiences may also help PLWH develop the knowledge, skills and confidence they need to manage their health. Unexpectedly, in our study, we found that a spouse’s HIV status was a key determinant of the PAM score; to the best of our knowledge, this finding has not been previously reported. The most likely explanation for this is that patients with an HIV-positive spouse are more likely to disclosure their HIV status at home, which may facilitate learning and discussion of HIV management between couples, thus enhancing their knowledge, skills and confidence, facilitating their engagement in their HIV care (37). Although the causal relationships between the associated factors and patient activation are likely to be complicated and not one-way, health-related behavioral interventions and screening potential patients may be bolstered by these factors.

We found higher PAM scores were associated with HIV viral suppression, which was mediated through gender, but they were not associated with CD4 count [that finding was partially supported by a previous study (13)]. Unexpectedly, this association was partly mediated by gender, which, to the best of our knowledge, has not been previously reported. The most likely explanation for this is that, as men take on more social and family responsibilities in Yi minority society, they have to leave their hometown frequently to make a living in big cities, which makes it harder to spend more time and money on their own health. Thus, they are more likely to miss the opportunity to receive healthcare services from local medical staff. Moreover, we found that PAM scores were not associated with CD4 count, which might be explained by the fact that CD4 counts are affected by numerous immune and nonimmune factors, such as tuberculosis (TB) co-infection, hepatitis B virus (HBV) co-infection and CD4 count at baseline (38), and therefore it serves as a crude indicator of HIV disease severity (13); On the other hand, this may have been due to the sample size was not large enough to detect the relationship between patient activation and CD4 count in low- and middle-income settings. Further study is needed to discover how patient activation impacts CD4 count.

Unexpectedly, inconsistent with the previous studies, our study did not find that the ART adherence mediated the relationship between patient activation and viral suppression. Overall, ART adherence was good among the Yi minority PLWH who participated in this study. This finding could be due to the fact that self-health management knowledge and skills (e.g., monitoring the effect of ART, consulting their healthcare providers), other than maintaining good adherence, were ignored by the Yi minority PLWH with low patient activation. Our findings indicate that simply maintaining good ART adherence is not enough for this lifelong illness, and tailored patient activation interventions are necessary to improve health outcomes in minority PLWH with low patient activation.

## Limitations

Our study has several limitations that should be considered when interpreting the results. First, we were unable to draw causal inferences from our study due to its cross-sectional design, because it was not possible to examine whether the detected associations persist over time. Although our findings identify several factors related to patient activation and the relationship between patient activation and HIV viral suppression, a longitudinal study is needed to explore the relationship accurately. Second, we primarily included participants engaged in care in an HIV treatment clinic; these participants had a better adherence to treatment and had access to professional medical consultation, education and care for their health management. However, it is likely that PLWH who are not receiving care have lower PAM scores and are more vulnerable, as they have less access to professional support. Thus, future studies should include these individuals to reinforce the observed relationships between patient activation and other variables. Third, there are considerable differences in terms of ethnicity, culture, local language and public health system among low- and middle-income regions. Thus, caution is needed when generalizing the findings of our study to other populations of PLWH (18). Fourth, this study was conducted in a Yi minority region, and the translation from Mandarin to Yi language may have led to inaccuracies. Finally, most of the data for this study were taken from patient self-reports. It is possible that the responses of participants were influenced by social desirability and recall bias. For the former, we adhered to our promise of anonymity of responses, which may have minimized potential problems with this type of bias; for the latter, prospective studies should be conducted in the future to balance the recall bias.

## Conclusion

Patient activation is poor in Yi minority PLWH, and the factors we identified included personal monthly income, self-rated method of ART effect, illness perception, having disease knowledge learning experience and spouse’s HIV status. Moreover, a higher PAM score was associated with viral suppression. Our findings will help healthcare providers systematically evaluate these factors to identify individuals with low patient activation. These research findings may also be used to develop interventions for minority PLWH in low- and middle-income regions to increase their involvement in the healthcare system, and they suggest that viral suppression may be improved by facilitating an increase in patient activation.

## Data availability statement

The raw data supporting the conclusions of this article will be made available by the authors, without undue reservation.

## Ethics statement

The Institutional Review Board of Xiangya Nursing School of Central South University approved this study (No. E2021007). During the study, all participants provided informed consent and participated voluntarily after a detailed explanation of the study aims. A written informed consent (or a thumb-printed consent, if the participant was illiterate) was required to enroll. The participants were not paid for their participation.

## Author contributions

WeA conducted the study design, data collection, analysis and interpretation of the data, and wrote the manuscript. XT, XX, and WaA contributed to the data collection, analysis, and interpretation, and commented on drafts. HW supervised the study design, data collection, analysis and interpretation of the data, and commented on the draft article. All authors contributed to this article and approved the submitted version.

## Funding

This work was supported by the Provincial Natural Science Foundation of Hunan Grant (No. 2022JJ30769); and the School-level Projects of Panzhihua University (No. 2021PY003).

## Conflict of interest

The authors declare that the research was conducted in the absence of any commercial or financial relationships that could be construed as a potential conflict of interest.

## Publisher’s note

All claims expressed in this article are solely those of the authors and do not necessarily represent those of their affiliated organizations, or those of the publisher, the editors and the reviewers. Any product that may be evaluated in this article, or claim that may be made by its manufacturer, is not guaranteed or endorsed by the publisher.
